# Beyond the Frontlines: Burnout Among Emergency Healthcare Providers in Jordan

**DOI:** 10.1155/jonm/9157658

**Published:** 2026-04-15

**Authors:** Mahmoud T. Alwidyan, Omaymah M. Algharaibeh, Mokhallad M. Aljanabi, Ala F. Ashour, Alaa O. Oteir

**Affiliations:** ^1^ Department of Allied Medical Sciences, Faculty of Applied Medical Sciences, Jordan University of Science and Technology, Irbid, Jordan, just.edu.jo; ^2^ Department of Physiology and Biochemistry, Faculty of Medicine, Jordan University of Science and Technology, Irbid, Jordan, just.edu.jo; ^3^ Department of Paramedicine, Monash University, Melbourne, Victoria, Australia, monash.ac.za

**Keywords:** burnout, Copenhagen Burnout Inventory, emergency healthcare providers, Jordan

## Abstract

**Background:**

Burnout among emergency healthcare providers, including physicians, nurses, and paramedics, is a growing global concern, particularly in developing countries where prevalence rates can reach 70%–80%. Burnout negatively impacts healthcare systems and patient outcomes. However, limited studies compare burnout rates across these professions or focus on the Middle Eastern context, including Jordan. This study aims to assess burnout prevalence among emergency physicians, nurses, and paramedics in Jordan and identify associated demographic and work‐related factors.

**Methods:**

A cross‐sectional descriptive study was conducted using a paper‐based, self‐administered questionnaire incorporating the Copenhagen Burnout Inventory (CBI). Participants were drawn from emergency departments in 15 hospitals and 13 ambulance stations. Data were analyzed using SPSS Version 25, employing descriptive statistics, ANOVA, and logistic regression to identify burnout predictors.

**Results:**

A total of 560 participants completed the survey (response rate: 70%). The prevalence rates for personal, work‐related, and patient‐related burnout were 83.7%, 79.2%, and 64.0%, respectively, with overall high burnout rates (75.6%). Sleep deprivation (*p* ≤ 0.002), poor supervisor relationships (*p* ≤ 0.01), and attempts to change workplaces (*p* ≤ 0.03) were associated with all burnout dimensions. Female participants and those with one to three children showed higher burnout odds (*p* = 0.004, *p* = 0.045, respectively). No significant differences in burnout rates were observed among professions.

**Conclusions:**

This study highlights alarmingly high burnout prevalence rates among emergency healthcare providers in Jordan, stressing the need for tailored strategies to mitigate burnout and improve the resilience of the healthcare system. Future research should explore the long‐term impacts of systemic factors and post–COVID‐19 dynamics.

**Contribution to Emergency Nursing Practice**
 What is already known about this topic?
 Studies have suggested high rates of burnout among healthcare providers. In the emergency healthcare setting, caring for patients in urgent needs is demanding and stressful, leading in many instances to burnout.
 What does this paper add to the currently published literature?
 This study is the first of its kind in Jordan and the region. The study findings reveal that emergency healthcare providers exhibit high rates of burnout. Interestingly, our study shows no significant differences in burnout rates based on the type of profession.
 What is the most important implication for clinical emergency nursing practice?
 There is a crucial need for specific interventions to address burnout among emergency healthcare providers in Jordan. Addressing burnout is paramount for sustaining a resilient and effective emergency healthcare system in the country.

## 1. Introduction

In the fast‐paced, high‐pressure world of emergency healthcare, the commitment of physicians, nurses, and paramedics is crucial for safeguarding human life. Frontline healthcare professionals, including emergency physicians, emergency nurses, and paramedics, collectively referred to as emergency healthcare providers, play a vital role in promptly responding to emergencies, ranging from accidents to disasters. However, the nature of their work often places them at significant risk of burnout [1–4].

Burnout is a state of physical and emotional exhaustion caused by prolonged exposure to various stressors, overwhelming workload, and a lack of appropriate support [5–7]. Studies in developed countries indicate that burnout rates among healthcare providers range from 30% to 50%, with emergency healthcare workers experiencing some of the highest rates [1, 8–10]. Alarmingly, studies in developing countries report even higher burnout rates, reaching up to 70%–80% in some contexts [11, 12]. The high rates of burnout could negatively impact the competency of the healthcare organizations and, in turn, patient outcomes [7, 13].

Burnout among emergency healthcare providers is particularly concerning due to the stressful nature of their work [4, 10]. These professionals work with critically ill and injured patients under significant time pressure, with minimal room for error [10, 14]. This demanding environment can lead to high levels of stress, with studies showing burnout prevalence rates as high as 65% for physicians, 70% for nurses, and 60% for paramedics in some settings [4, 7, 15].

In Jordan, a lower‐middle‐income country, the healthcare system encounters unique challenges, including resource constraints, high patient‐to‐provider ratios, and prolonged waiting times [16, 17]. Emergency departments, in particular, are often characterized by overcrowding and long working hours. Furthermore, healthcare providers in Jordan, especially in emergency settings, are increasingly exposed to workplace violence, which has been identified as a significant occupational hazard [18]. These systemic and environmental factors create a high‐stress workplace that may uniquely contribute to burnout risk among Jordanian emergency healthcare workers, beyond the universal stressors of emergency care [19–21].

Although emergency physicians, emergency nurses, and paramedics work under similar conditions, providing direct and urgent care to patients in need, they have different scopes of practice, work‐related stressors, and potentially different burnout rates [3, 10]. Many studies have investigated rates of burnout among physicians [5, 22], nurses [15, 23], and paramedics [1–3] separately, but few have directly compared them. Furthermore, most research focuses on specific factors such as demographic characteristics, work conditions, and experiences [5, 24–26], with limited studies addressing burnout in emergency healthcare workers in Jordan, particularly among paramedics. This study aims to fill this gap by assessing burnout rates among emergency physicians, emergency nurses, and paramedics in Jordan, identifying associated factors, and proposing strategies to mitigate and prevent burnout in this unique healthcare context.

## 2. Methods

### 2.1. Design

This was a cross‐sectional descriptive study assessing the rates of burnout among frontline healthcare providers in Jordan using a paper‐based questionnaire.

### 2.2. Study Setting

Jordan is a lower‐middle‐income country with a healthcare system that encompasses four key sectors: the Ministry of Health (30 public hospitals), Royal Medical Services (RMS) (12 hospitals), the private sector (68 hospitals), and university hospitals (2 hospitals) [27]. The ambulance service in Jordan is exclusively provided by the Jordan Civil Defense. The ambulance service providers include paramedics, nurses, and emergency medical technicians (EMTs) [28]. For the purposes of this study, 15 hospitals were conveniently selected from the public, RMS, and private sectors along with 13 ambulance stations affiliated with the Jordan Civil Defense.

### 2.3. Instrument

Data were collected using a self‐administered questionnaire. This questionnaire was anonymous and included 36 items addressing three parts. The first part includes the demographic characteristics (9 items). The second part includes work condition questions (8 items). The third part includes the Copenhagen Burnout Inventory (CBI), which encompasses 19 questions divided into three domains of burnout: personal burnout (6 items), work‐related burnout (7 items), and patient‐related burnout (6 items) [29]. Each of the three dimensions was scored separately. The scoring system ranges from 0% to 100%, where 0% represents “*Never*,” 25% “*Seldom*,” 50% “*Sometimes*,” 75% “*Often*,” and 100% “*Always*.” According to the authors’ instructions, the score for each domain is the mean score for that domain, and an average score of 50 or higher denotes the presence of burnout. On a scale of 0–100, a score of 50 or above signifies a moderate level of burnout, and a score of 75 or above signifies a high level of burnout. This cutoff point of ≥ 50 is well established in the literature and has been widely used in previous studies to define burnout, facilitating comparability across different populations and settings [8, 30, 31]. The validity and reliability of the CBI tool have been previously assessed [29]. Cronbach’s alpha values of personal burnout, work‐related burnout, and patient‐related burnout dimensions were 0.87, 0.83, and 0.86, respectively, indicating good internal consistency.

### 2.4. Sampling Strategy and Participants

The sample was drawn from the emergency departments of 15 hospitals including eight governmental, three private, and two military hospitals. Paramedics and nurses were also recruited from 13 ambulance stations. A multistage sampling strategy was employed. First, hospitals and ambulance stations were purposively selected to ensure representation from all key healthcare sectors in Jordan: the Ministry of Health (public), RMS (military), the private sector, and the Civil Defense (ambulance service). Within these selected locations, a convenience sampling method was used for individual participants; questionnaires were distributed to all eligible physicians, nurses, and paramedics present during the data collection visits, allowing them the opportunity to participate. Participation in the study was voluntary and anonymous, conducted only after obtaining the necessary approvals. Eligible participants included emergency department physicians, nurses, and paramedics actively working in the selected hospitals. For the ambulance service, eligibility was limited to paramedics or nurses employed by the Civil Defense.

We employed the Raosoft online software to estimate the required sample size for our study, aiming for a confidence level of 95% and an accuracy level (confidence interval [CI]) of 5%. Based on these levels, the minimum number of emergency healthcare workers necessary to carry out this study was 385 [32]. A total of 800 questionnaires were distributed to potential participants including physicians and nurses working in the emergency departments of military, private, and governmental hospitals as well as paramedics from the stations of the Jordanian Civil Defense.

### 2.5. Data Analysis

Descriptive statistics were utilized to describe participants’ demographic characteristics and factors that influence burnout, using means and standard deviations for continuous variables and frequencies and percentages for categorical variables. The CBI continuous variables were converted into dichotomous variables at a cutoff point of 50 (i.e., having burnout and not having burnout) [8, 30, 31]. Analysis of variance (ANOVA) was used to assess the association between continuous variables. Binary logistic regression was conducted to identify the predictors of burnout. Univariate and multivariate logistic regression models were applied for each burnout dimension. The 95% CIs and odds ratios were calculated, with a *p* value < 0.05 to determine statistical significance. All data were analyzed using SPSS Version 25 (Chicago, Illinois) [33].

## 3. Results

The returned questionnaires totaled 560, yielding a response rate of 70.0%. Table 1 describes the demographic characteristics of the study participants. The mean age of the participants was 30.5 (SD ± 5.8) years, with a majority being male (58.6%). Among the professionals surveyed, nurses comprised the largest group (47.9%), followed by paramedics (35.2%) and physicians (16.8%). The majority of participants were married (60.3%) and held a bachelor’s degree (53.4%). In terms of employment, a majority worked for the public (41%) and had one to 5 years of experience (34.6%).

**TABLE 1:** Demographic characteristics of study participants.

Variable		*n* (%)
Age	Mean (SD)	30.5 (5.8)

Sex	Male	327 (58.6)
	Female	231 (41.4)

Marital status	Single	200 (36.2)
	Married	333 (60.3)
	Others (not specified)	19 (3.4)

Number of children	None	260 (46.9)
	1–3	247 (44.6)
	More than 3	47 (8.5)

Profession	Physician	94 (16.8)
	Nurse	268 (47.9)
	Paramedics	197 (35.2)

Educational level	Diploma or below	200 (36.3)
	Bachelor	295 (53.4)
	Graduate studies	57 (10.3)

Employer	Public hospitals	229 (41.0)
	RMS hospitals	76 (13.6)
	Civil defense	169 (30.2)
	Private hospitals	85 (15.2)

Monthly income (JD)	400 or less	267 (48.0)
	401–600	159 (28.6)
	601–800	77 (13.8)
	801 or above	53 (9.5)

Experience	< 1 year	34 (6.1)
	1–5 years	193 (34.6)
	6–10 years	144 (25.9)
	> 10 years	186 (33.4)

*Note:* n (%): number (percentage); JD: Jordan dinar (1 JD = 1.41 United States Dollar); SD: standard deviation.

### 3.1. Burnout Prevalence Rates

The mean scores for the CBI dimensions, stratified by participants’ profession, were assessed. As shown in Table 2, the mean scores for burnout in the three dimensions were 66.8 (±18.9) for personal burnout, 64.7 (±19.5) for work‐related burnout, and 56.3 (±22.2) for patient‐related burnout. The ANOVA test was used to assess the association between burnout dimensions and the type of profession. Results indicated no significant differences between participants’ professions in all burnout dimensions, including personal burnout (*p* = 0.70), work‐related burnout (*p* = 0.35), and patient‐related burnout (*p* = 0.11).

**TABLE 2:** Descriptive statistics of burnout dimensions across professional type.

Burnout dimension	Total (*n* = 560) M (SD)	Physicians (*n* = 94) M (SD)	Nurses (*n* = 268) M (SD)	Paramedics (*n* = 197) M (SD)	*p*‐value^∗^	Percentage burnout (overall) (Score ≥ 50)
Personal burnout	66.8 (18.93)	65.7 (19.6)	67.5 (18.0)	66.4 (19.8)	0.70	83.7%
Work burnout	64.7 (19.52)	67.4 (17.7)	64.4 (19.6)	63.6 (20.1)	0.35	79.2%
Patient burnout	56.3 (22.25)	59.9 (21.9)	56.6 (21.5)	54.0 (23.2)	0.11	64.0%
						75.6%

*Note:* M: mean; *n*: sample size.

Abbreviation: SD: standard deviation.

^∗^ANOVA test.

Table 3 illustrates the association between burnout and the demographic characteristics. Significant associations were observed across all burnout dimensions. For personal burnout, participants with 1–3 children were more likely to report burnout (88.6%) compared to those with no children (80.5%) or more than three children (73.9%) (*p* = 0.011). For work‐related burnout, those with less than 1 year of experience had lower rates of burnout (71.0%) compared to those with 6–10 years of experience (87.0%, *p* = 0.048).

**TABLE 3:** Association between burnout and the demographic characteristics.

Variable		Personal burnout (score ≥ 50)	*p*‐value^∗^	Work‐related burnout (score ≥ 50)	*p*‐ value	Patient‐related burnout (score ≥ 50)	*p*‐value
Overall		83.7%		79.2%		64.0%	

Age	29 or less	183 (80.6)	0.19	179 (77.5)	0.68	135 (58.4)	**0.04**
	30–39	228 (86.4)		208 (80.6)		184 (69.2)	
	40 or above	31 (79.5)		28 (77.8)		23 (62.2)	

Gender	Male	190 (84.4)	0.66	171 (78.1)	0.35	128 (56.9)	**0.002**
	Female	259 (83.0)		249 (79.8)		219 (69.1)	

Marital status	Single	159 (81.1)	0.24	149 (78.0)	0.54	119 (61.0)	0.47
	Married	272 (85.3)		255 (80.4)		214 (66.3)	
	Others	16 (94.1)		12 (70.6)		11 (61.1)	

Number of children	None	202 (80.5)	**0.01**	192 (75.9)	0.14	160 (63.2)	0.56
	1–3	209 (88.6)		195 (83.0)		158 (65.8)	
	More than 3	34 (73.9)		31 (75.6)		26 (57.8)	

Profession	Physician	76 (84.4)	0.91	77 (84.6)	0.37	64 (68.1)	0.38
	Nurse	221 (84.0)		199 (78.0)		171 (65.0)	
	Paramedics	153 (82.7)		145 (78.0)		112 (60.2)	

Educational level	Diploma or below	153 (81.4)	0.33	141 (77.0)	0.51	117 (61.9)	0.83
	Bachelor	242 (84.3)		226 (79.0)		187 (64.5)	
	Graduate studies	51 (89.5)		48 (84.2)		37 (64.9)	

Employer	Public hospitals	196 (88.7)	0.05	185 (84.1)	0.10	160 (71.1)	**0.02**
	RMS hospitals	58 (77.3)		52 (75.4)		48 (64.0)	
	Civil defense	128 (81.5)		122 (77.2)		93 (58.9)	
	Private hospitals	68 (80.0)		62 (72.9)		46 (54.1)	

Monthly income (JD)	400 or less	213 (82.6)	0.54	195 (76.8)	0.50	164 (63.1)	0.84
	401–600	128 (84.8)		123 (82.0)		96 (63.2)	
	601–800	65 (89.0)		60 (83.3)		52 (68.4)	
	801 or above	43 (81.1)		42 (79.2)		34 (65.4)	

Experience	< 1 year	25 (73.5)	0.12	22 (71.0)	**0.048**	19 (57.6)	0.76
	1–5 years	150 (81.1)		142 (75.5)		122 (63.5)	
	6–10 years	121 (88.3)		120 (87.0)		93 (66.9)	
	> 10 years	153 (85.0)		136 (78.6)		112 (63.3)	

*Note:* Bold values indicate significant results.

Abbreviation: JD: Jordan dinar.

^∗^Chi‐square test was used.

Patient‐related burnout was associated with age, gender, and employer type. Participants aged 30–39 years reported higher rates (69.2%) compared to those aged 29 or less (58.4%, *p* = 0.04). Females had significantly higher rates (69.1%) than males (56.9%, *p* = 0.002). Additionally, individuals employed in public hospitals experienced higher rates of patient‐related burnout (71.1%) compared to those in private hospitals (54.1%, *p* = 0.02).

Table 4 shows significant associations between work conditions and burnout across its three dimensions. For personal burnout, sleep deprivation (87.6% vs. 64.0%, *p* < 0.001), attempting to change workplaces (89.6% vs. 73.8%, *p* < 0.001), and poor relationships with supervisors (93.4% vs. 80.9%, *p* < 0.001) were significantly associated with higher burnout rates.

**TABLE 4:** Association between burnout and work conditions.

Variable		Personal burnout (score ≥ 50)	*p* value^∗^	Work‐related burnout (score ≥ 50)	*p* value	Patient‐related burnout (score ≥ 50)	*p* value
Overall		83.7%		79.2%		64.0%	
Do you work shifts at your job?	Yes	324 (84.6)	0.13	313 (80.9)	0.04	257 (65.6)	0.09
	No	116 (80.0)		100 (73.0)		83 (58.9)	
How many hours is the length of the shifts?	8 h	237 (84.6)	0.67	214 (76.7)	0.13	180 (63.2)	0.12
	12 h	43 (84.3)		37 (72.5)		28 (52.8)	
	16 h	47 (88.7)		46 (88.5)		37 (69.8)	
	24 h	41 (80.4)		38 (80.9)		30 (60.0)	
	> 24 h	69 (80.2)		74 (85.1)		63 (73.3)	
How many hours do you sleep per day?	< 4	56 (93.3)	**<** **0.001**	52 (85.2)	**0.004**	43 (70.5)	0.32
	4–6	248 (88.3)		227 (84.1)		187 (66.1)	
	7–8	121 (72.9)		120 (70.6)		100 (59.5)	
	> 8	25 (86.2)		22 (75.9)		17 (58.6)	
Do you feel that you suffer from sleep deprivation?	Yes	395 (87.6)	**<** **0.001**	371 (83.2)	**<** **0.001**	307 (67.5)	**<** **0.001**
	No	55 (64.0)		50 (58.8)		40 (46.0)	
Is there any possibility to leave your current job in the next 12 months?	No	191 (77.3)	**0.001**	179 (72.2)	**<** **0.001**	145 (57.1)	**0.001**
	Low	106 (86.2)		99 (83.2)		86 (70.5)	
	Moderate	86 (91.5)		83 (88.3)		67 (71.3)	
	High	57 (93.4)		54 (90.0)		43 (71.7)	
Have you tried to change your workplace?	Yes	301 (89.6)	**<** **0.001**	287 (86.7)	**<** **0.001**	238 (70.2)	**<** **0.001**
	No	144 (73.8)		129 (66.5)		104 (53.1)	
Do you have a good relationship with your supervisor?	Yes	335 (80.9)	**<** **0.001**	309 (75.9)	**<** **0.001**	256 (61.1)	**0.006**
	No	114 (93.4)		111 (90.2)		90 (73.8)	
Do you have a good relationship with the majority of coworkers?	Yes	402 (82.9)	**0.01**	375 (78.0)	**0.002**	312 (63.0)	**0.03**
	No	44 (84.0)		43 (95.6)		33 (78.6)	

*Note:* Bold values indicate significant results.

^∗^Chi‐square test was used.

For work‐related burnout, shift work (80.9% vs. 73.0%, *p* = 0.04), sleep deprivation (83.2% vs. 58.8%, *p* < 0.001), high turnover intentions (90.0% vs. 72.2%, *p* < 0.001), attempting to change workplaces (86.7% vs. 66.5%, *p* < 0.001), and poor relationships with supervisors (90.2% vs. 75.9%, *p* < 0.001) and coworkers (95.6% vs. 78.0%, *p* = 0.002) were significantly associated with higher burnout rates.

For patient‐related burnout, significant associations were found with sleep deprivation (67.5% vs. 46.0%, *p* < 0.001), high turnover intentions (71.7% vs. 57.1%, *p* = 0.001), attempting to change workplaces (70.2% vs. 53.1%, *p* < 0.001), and poor relationships with supervisors (73.8% vs. 61.1%, *p* = 0.006) and coworkers (78.6% vs. 63.0%, *p* = 0.03).

### 3.2. Predictors of Burnout

A backward stepwise logistic regression analysis was conducted to identify significant predictors of burnout. Table 5 presents the findings of the three CBI dimensions of burnout. Variables were removed iteratively using a threshold of *p* > 0.05.

**TABLE 5:** Predictors of CBI burnout dimensions.

Variable	Personal burnout OR (95% CI)	*p* value	Work‐related burnout OR (95% CI)	*p* value	Patient‐related burnout OR (95% CI)	*p* value
Gender (female)	—	—	—	—	1.87 (1.23–2.90)	0.004
Number of children	—	—	—	—	—	—
None	—	0.045	—	—	—	—
1–3	1.86 (1.02–3.38)	0.04	—	—	—	—
More than 3	0.68 (0.28–1.67)	0.40	—	—	—	—
Suffer from sleep deprivation (Yes)	2.90 (1.49–5.69)	0.002	3.44 (1.95–6.07)	< 0.001	2.80 (1.65–4.73)	< 0.001
Tried to change workplace (Yes)	2.77 (1.59–4.83)	< 0.001	2.76 (1.68–4.52)	< 0.001	1.58 (1.05–2.40)	0.03
Have a good relationship with the supervisor (No)	4.17 (1.50–11.61)	0.006	3.53 (1.56–7.96)	0.002	2.02 (1.17–3.5)	0.01

Abbreviations: CI, confidence interval; JD, Jordan dinar; OR, odds ratio; RMS, Royal Medical Services.

The odds of personal burnout were 1.8 times higher for participants with one to three children compared to those without children (OR = 1.86, 95% CI = 1.02–3.38, *p* = 0.04). Similarly, participants experiencing sleep deprivation had 2.9 times higher odds of personal burnout (OR = 2.90, 95% CI = 1.49–5.69, *p* = 0.002). The odds were also 2.7 times higher for participants who had attempted to change their workplace (OR = 2.77, 95% CI = 1.59–4.83, *p* < 0.001), and 4.1 times higher for those reporting poor relationships with their supervisors (OR = 4.17, 95% CI = 1.50–11.61, *p* = 0.006).

For work‐related burnout, participants experiencing sleep deprivation had 3.4 times higher odds compared with participants with no sleep deprivation (OR = 3.44, 95% CI = 1.95–6.07, *p* < 0.001). Attempting to change workplaces also significantly increased the odds of work‐related burnout by 2.8 times (OR = 2.76, 95% CI = 1.68–4.52, *p* < 0.001). Furthermore, poor relationships with supervisors were associated with 3.5 times higher odds of work‐related burnout (OR = 3.53, 95% CI = 1.56–7.96, *p* = 0.002).

For patient‐related burnout, female participants had 1.9 times higher odds compared to male participants (OR = 1.87, 95% CI = 1.23–2.90, *p* = 0.004). Sleep deprivation increased the odds of patient‐related burnout by 2.8 times (OR = 2.80, 95% CI = 1.65–4.73, *p* < 0.001). Similarly, participants who attempted to change workplaces had 1.6 times higher odds of patient‐related burnout (OR = 1.58, 95% CI = 1.05–2.40, *p* = 0.03). Poor relationships with supervisors were associated with 2.0 times higher odds of patient‐related burnout (OR = 2.02, 95% CI = 1.17–3.50, *p* = 0.01).

## 4. Discussion

The aim of this study was to assess the prevalence rates of burnout among healthcare providers working in emergency settings including emergency physicians, emergency nurses, and paramedics in Jordan and explore potential associated factors. The results reveal that emergency healthcare providers in Jordan exhibit high rates of burnout. No significant variations in burnout rates were observed based on participants’ profession. However, burnout rates varied based on sex, number of children, sleep deprivation, workplace change, and relationships with supervisors.

The prevalence rates of burnout among the emergency healthcare providers in Jordan are among the highest reported in the literature, posing a significant concern for the healthcare system. Professionals working in emergency settings showed high rates of personal burnout (83.7%) and work‐related burnout (79.2%), as well as moderate rates of patient‐related burnout (64.0%), resulting in an overall high burnout rate (75.6%). These elevated rates can be attributed to the challenging working conditions in emergency settings where healthcare providers deal with critically ill and injured patients under time pressure [10, 14, 34].

Prior studies in Jordan have also highlighted high rates of burnout among healthcare providers. For instance, Hamaideh [26] found high rates of emotional exhaustion, moderate rates of depersonalization, and moderate rates of personal accomplishment among mental health nurses using the Maslach Burnout Inventory (MBI) [26]. Another study comparing burnout between nurses and teachers in Jordan reported moderate rates of burnout among nurses, with higher rates compared to teachers [23]. However, these studies utilized the MBI, which assesses burnout from various angles through a comprehensive method, whereas the CBI primarily concentrates on fatigue and exhaustion, aligning more closely with the definition of burnout. A more recent study assessed burnout among medical residents in Jordan using the CBI approach and found high rates of burnout among medical residents in Jordan (77.5%), which are similar to the findings in our study [27].

Interestingly, the present study found no statistically significant differences in burnout prevalence across professional groups. A plausible explanation for this finding is that the systemic pressures inherent within the Jordanian emergency care system are so pervasive and intense that they obscure potential professional role variations. Common stressors, such as resource limitations, high patient acuity, overcrowded facilities, and frequent exposure to workplace violence [16, 18, 19], likely foster a uniformly high‐stress environment where the specific professional role becomes less of a determinant of burnout risk. This observation underscores that burnout in this setting represents a systemic rather than profession‐specific phenomenon, necessitating comprehensive, organization‐wide interventions aimed at structural reform and workforce support. However, it is also important to acknowledge that the uneven sample sizes across the three professional groups (with nurses comprising the largest group) may have influenced the statistical power to detect significant differences, a point we address in the limitations section.

Our study is the first to assess burnout rates among paramedics in Jordan, which are comparable with those of emergency physicians and emergency nurses. While the majority of paramedics on our sample work in the Civil Defense, some of them work in hospitals’ emergency departments in Jordan. A study performed in Poland found that paramedics showed the lowest rates of burnout compared to physicians and nurses [2]. Additionally, a study in Germany found 20%–40% rates of high burnout among emergency medical service (EMS) workers [1]. A systematic review on prevalence rates of burnout among paramedics reported a range between 16% and 56% [3]. These findings contradict our study findings. The higher rates observed in our study need further investigation and may be attributed to the quasimilitary style of the fire‐based EMS and the extended working hours of EMS providers in Jordan.

When comparing burnout rates internationally, Jordan surpasses Western countries, with reported rates of 40% in the United States [35], 65% in Romania [36], and 42.1% in New Zealand [8]. This discrepancy could be elucidated by the nature of the emergency care environment in Jordanian hospitals, characterized by long working hours and the prevalent issue of violence against emergency teams, both of which may contribute to the higher overall burnout rates [5, 37, 38]. Regionally, a systematic review in the Middle East reported burnout rates between 40% and 60%, with the majority of studies focusing on physicians and nurses [39]. Research studies that defined overall burnout as the presence of burnout in at least one of its categories reported higher rates, with 84% in the United Arab Emirates (UAE) [11] and 70% in Saudi Arabia [12]. In contrast, studies that defined overall burnout as the presence of burnout in all three categories showed lower rates, such as 11.7% in Yemen [6], 12.6% in Qatar [5], and 6.3% in Saudi Arabia [40].

### 4.1. Predictors of Burnout

Our study indicated that being female nearly doubles the likelihood of experiencing patient‐related burnout. Similar findings have been reported in previous studies, which suggest that females tend to experience higher rates of burnout than males [4, 13, 22, 30]. Additionally, having one to three children increased the odds of experiencing personal burnout by 1.8 times. This finding aligns with a study on Japanese nurses [41] but contradicts other studies that suggested having children protects nurses from experiencing burnout [42] and enhances personal accomplishment [43].

Sleep deprivation was associated with all three dimensions of burnout: personal, work‐related, and patient‐related. While our study found no significant effect of working shifts or working hours on burnout, sleep deprivation was associated with personal burnout, consistent with previous studies [24, 37].

Our finding that poor relationships with supervisors were strongly associated with all three burnout dimensions is critically important and has direct implications for nursing management. This aligns with growing evidence from Jordan and internationally that highlights the crucial role of leadership in shaping the work environment and staff well‐being [21, 44, 45]. For instance, research has shown that ethical leadership by nurse managers is negatively associated with counterproductive work behaviors among Jordanian nurses [21], while authentic leadership is linked to a stronger safety climate [45]. Our findings suggest that a lack of such positive, supportive leadership may be a key driver of burnout. This underscores an urgent need for healthcare organizations to invest in leadership development programs that equip managers with the skills for supportive supervision, empathetic communication, conflict resolution, and ethical decision‐making, thereby fostering a more respectful and psychologically safe work environment.

The challenging work environment in Jordanian emergency healthcare, including the threat of workplace violence, likely contributes to increased burnout. A recent thematic analysis conducted in Jordanian psychiatric hospitals indicated that inadequate staffing, poor security, and limited organizational support were key factors that fuel violence against nurses [19]. Although our setting differs, emergency departments in Jordan face comparable systemic issues. The chronic stress of operating in an environment where violence is perceived as a real threat, combined with limited managerial support, as indicated in our study, creates a perfect environment for burnout to thrive [21].

While our data are prepandemic, the findings of Algunmeeyn et al. [46] during COVID‐19 confirmed that factors such as fear of infection and perceived organizational support were significantly associated with burnout among Jordanian healthcare providers [46]. This indicates that the systemic vulnerabilities we identified, such as poor supervisory support, were perhaps amplified during the pandemic. This interpretation is further supported by a recent global meta‐analysis, which found a pooled burnout prevalence of 59.5% among nurses during the COVID‐19 pandemic, highlighting how crisis conditions can dramatically elevate burnout risk across diverse healthcare settings [47]. Future research may explore how these preexisting stressors interacted with the unique pressures of the health crisis.

To the best of our knowledge, this study is the first to assess rates of burnout among all emergency healthcare providers including physicians, nurses, and paramedics in Jordan and the region. This is also the first study to use the CBI in this cohort of emergency healthcare professionals in Jordan and the region. This research seeks to contribute to the global discourse on burnout and to provide actionable insights tailored to the needs of emergency healthcare providers.

### 4.2. Implications for Emergency Nursing

The findings of this study underscore the urgent need for targeted interventions by nursing management and hospital administration. The critical role of professional relationships, both with supervisors and coworkers, highlights the necessity for a broader cultural shift within healthcare institutions. Studies in Jordan have demonstrated that negative environments, such as those characterized by workplace incivility, can have detrimental effects even in educational settings [48].

To address the critical role of poor supervisory relationships, we recommend that healthcare organizations invest in structured, evidence‐based leadership training programs for nurse managers and supervisors. These programs should focus on building skills in supportive supervision, empathetic communication, conflict resolution, and ethical leadership, skills directly linked to reducing burnout and improving team outcomes [21, 44, 45]. Healthcare organizations should also hold regular, structured debriefing sessions after critical incidents to help reduce stress and support staff well‐being.

In the context of workplace violence, institutions must create and rigorously enforce clear, zero‐tolerance policies that are strongly supported by management. This includes implementing enhanced security measures, establishing transparent and nonpunitive incident reporting mechanisms, and providing immediate support for affected staff.

To address sleep deprivation and exhaustion, hospital administrators should review and revise shift scheduling policies to ensure that reasonable working hours and adequate rest periods are actively practiced, not merely documented. This may involve limiting consecutive long shifts and ensuring access to rest facilities.

Furthermore, the critical role of professional relationships highlights the necessity for a broader cultural shift within healthcare institutions. Interventions should not only focus on individuals but also aim to improve the overall organizational culture by promoting civility, teamwork, and mutual respect at all levels. Healthcare organizations should implement regular, structured debriefing sessions after critical incidents to provide a safe space for staff to process experiences, reduce stress, and foster peer support.

### 4.3. Limitations

This study has several limitations. The inherent nature of a cross‐sectional design precludes any causal inferences; the associations identified should be interpreted as relationships rather than causal pathways. Additionally, the use of a self‐administered questionnaire may be subject to response bias. While we used a multistage sampling approach, the selection of hospitals and stations was purposive, and participants within those sites were recruited via convenience sampling. It is possible that settings not included in this study have different working conditions and, consequently, different burnout outcomes, which may limit the generalizability of our findings. The uneven sample sizes across professional groups (with nurses being the largest group) may have limited our ability to detect statistically significant differences between professions.

The dichotomization of burnout scores at the ≥ 50 cutoff, while enhancing clinical interpretability and comparability with other studies, may result in a loss of nuanced information compared to analyzing burnout as a continuous variable. Finally, it is important to acknowledge that data were collected prior to the COVID‐19 pandemic. While our findings provide a valuable prepandemic baseline, burnout patterns may have evolved due to the unprecedented pressures of the health crisis. Therefore, the generalizability of these findings to the current postpandemic context may be limited, and future research is needed to explore the long‐term impacts of the pandemic on burnout among emergency healthcare providers in Jordan.

## 5. Conclusion

This study reveals alarmingly high burnout prevalence rates among emergency healthcare providers in Jordan, surpassing international averages. Emergency physicians, nurses, and paramedics experience significant personal, work‐related, and patient‐related burnout, which was associated with factors such as sleep deprivation, poor supervisory relationships, and intentions to change workplaces. Female providers and those with one to three children are particularly at risk for specific burnout dimensions, highlighting the unique challenges faced by different demographic groups. These findings suggest that the Jordanian emergency care setting may exacerbate burnout, with adverse implications for both provider well‐being and patient care.

As the first study to assess burnout across all emergency healthcare roles in Jordan using the CBI, this research provides a critical foundation for future policy and well‐being initiatives. The findings highlight that Jordan’s emergency care setting, characterized by systemic stressors, exacerbates burnout, adversely impacting both provider well‐being and patient care. Interventions to improve safety, reduce workloads, and enhance managerial support are urgently needed. Given that our data represent a prepandemic baseline, future research should explore the long‐term impacts of the COVID‐19 pandemic and postpandemic dynamics on burnout. Additionally, qualitative studies could offer deeper insights into workplace challenges and inform tailored strategies to mitigate burnout and sustain a resilient healthcare system.

## Author Contributions

Mahmoud T. Alwidyan: conceptualization, data curation, validation, formal analysis, funding acquisition, methodology, project administration, resources, and writing–original draft. Omayamah M. Algharaibeh: conceptualization, data curation, validation, formal analysis, methodology, and writing–original draft. Mokhallad M. Aljanabi: conceptualization, formal analysis, methodology, validation, and writing–review and editing. Ala F. Ashour: methodology, validation, and writing–review and editing. Alaa O. Oteir: conceptualization, formal analysis, methodology, validation, and writing–review and editing.

## Funding

This project was funded by Jordan University of Science and Technology (Grant number: 20190222).

## Ethics Statement

The Institutional Review Board (IRB) at Jordan University of Science and Technology reviewed and approved the study procedure (IRB No. 14/122/2019). Additionally, approvals were obtained from the Ministry of Health and the Jordanian Civil Defense before starting data collection. Participants who agreed to participate in the study were asked to sign a consent form that was included in the survey.

## Conflicts of Interest

The authors declare no conflicts of interest.

## Data Availability

Data will be available upon reasonable request.
